# Comparison of imputation variance estimators

**DOI:** 10.1177/0962280214526216

**Published:** 2014-03-28

**Authors:** RA Hughes, JAC Sterne, K Tilling

**Affiliations:** School of Social and Community Medicine, University of Bristol, Bristol, UK

**Keywords:** bootstrap confidence intervals, imputation inference, missing data, multiple imputation, variance estimator

## Abstract

Appropriate imputation inference requires both an unbiased imputation estimator and an unbiased variance estimator. The commonly used variance estimator, proposed by Rubin, can be biased when the imputation and analysis models are misspecified and/or incompatible. Robins and Wang proposed an alternative approach, which allows for such misspecification and incompatibility, but it is considerably more complex. It is unknown whether in practice Robins and Wang’s multiple imputation procedure is an improvement over Rubin’s multiple imputation. We conducted a critical review of these two multiple imputation approaches, a re-sampling method called full mechanism bootstrapping and our modified Rubin’s multiple imputation procedure via simulations and an application to data. We explored four common scenarios of misspecification and incompatibility. In general, for a moderate sample size (*n* = 1000), Robins and Wang’s multiple imputation produced the narrowest confidence intervals, with acceptable coverage. For a small sample size (*n* = 100) Rubin’s multiple imputation, overall, outperformed the other methods. Full mechanism bootstrapping was inefficient relative to the other methods and required modelling of the missing data mechanism under the missing at random assumption. Our proposed modification showed an improvement over Rubin’s multiple imputation in the presence of misspecification. Overall, Rubin’s multiple imputation variance estimator can fail in the presence of incompatibility and/or misspecification. For unavoidable incompatibility and/or misspecification, Robins and Wang’s multiple imputation could provide more robust inferences.

## 1 Introduction

Multiple imputation (MI)^1^ is the most commonly used technique for analysing incomplete data, which are frequently encountered in health-related research. Imputations are repeatedly and independently drawn from the posterior predictive distribution of the missing data given the observed data, under a Bayesian model, to generate m(≥2) imputed datasets. These imputed datasets are then analysed separately using complete data methods, and the *m* sets of results are combined using a simple set of rules. Appropriate imputation inference requires both an unbiased imputation estimator and an unbiased variance estimator.

MI was first developed for analysing large incomplete public shared datasets, for which there may be many analysts, with a wide range of scientific questions, who may not have the specialized knowledge required to generate statistically valid inferences when data are incomplete. It was envisioned that the task of generating multiple imputed datasets would be undertaken by someone with specialized knowledge of missing data methods.^2^ The analysts need only apply standard complete data statistical procedures to each imputed dataset separately and then combine the multiple estimates according to Rubin’s rules.^1^

At the time of imputation, it may not be possible to anticipate all analysis procedures that will be applied to the imputed datasets. Consequently, the assumptions of a given analysis procedure may differ to those of the imputation model. We define an analysis procedure to be incompatible with an imputation model when one or more assumptions of the imputation model contradict with those made by the analysis procedure. For example, the imputer may assume that two subgroups have the same population mean of an incomplete variable (where missingness is independent of subgroup status) whilst the analyst estimates the population mean in one subgroup only.

As with any modelling or statistical procedure, the imputation model may be misspecified; that is, the imputation model’s assumptions about the missing data mechanism (MDM) or the complete data are incorrect. For example, the imputation model could incorrectly assume homoscedastic errors. Similarly, for the analysis procedure.

The MI literature has focused primarily on generating methods and guidelines for reducing the bias of the imputation estimator. However, correct imputation inference also requires an unbiased imputation variance estimator and an efficient interval estimator with at least nominal coverage. In certain settings of misspecification and incompatibility of the models, for an unbiased imputation estimator, Rubin’s variance estimator was either upwardly or downwardly biased, which led to conservative or anti-conservative confidence intervals, respectively.^2–7^

There are alternatives to Rubin’s MI. Robins and Wang have proposed imputing under a frequentist procedure,^5^ that fixes the imputation model parameters at the observed data maximum likelihood estimate, and an imputation variance estimator that allows for misspecification and incompatibility of the imputation model and the analysis procedure, and for non- or semi-parametric analysis procedures. However, it is unclear whether the Robins and Wang MI procedure is an improvement over Rubin’s MI in situations typical of the use of imputation in practice. Also, the Robins and Wang imputation variance estimator is considerably more complex to compute than Rubin’s and importantly is more technically difficult for the analyst, although the greater burden of calculating the variance estimator is still placed on the imputer. With ever faster computers, a simpler solution could be to calculate the variance of the imputation estimator using re-sampling methods. Full mechanism bootstrapping (FMB) is a bootstrapping approach to imputation that can be applied to parametric problems.^8,9^

In this paper, we have conducted the first comparative evaluation of Rubin’s MI, our modified version of Rubin’s MI, Robins and Wang’s MI and FMB with respect to variance estimation and interval estimation. We have compared these four methods of imputation inference in four scenarios of incompatibility and misspecification of the imputation and analysis models that can occur in practice, via a data example (see Sections 2 and 4) and simulations (see Sections 5 and 6). In Section 2, we describe a motivating example for seeking an alternative to Rubin’s MI. Section 3 describes all four methods considered in the context of a specific example. Section 4 revisits the motivating example, applying these methods to data from the British Child and Adolescent Mental Health Study 1999 (B-CAMHS99).^10^ In Sections 5 and 6, we present our simulation study and conclude with a general discussion in Section 7.

## 2 Motivating example

There are several examples in the literature, where observations used for estimation at the imputation stage of MI are not used or available at the analysis stage. For example, confidential information may be used to improve estimation of the imputation model but cannot be disseminated to the analysts. Or, when external data are used to account for measurement error using MI and the external data are not included at the analysis stage because it may not be representative of the target sample.^11^ We briefly describe a case study where external data were used to impute measurements, which were not collected for any of the subjects of the target study.

The B-CAMHS99 measured conduct, hyperactive and emotional problems in 15 year olds using the strengths and difficulties questionnaire (SDQ).^12^ Researchers wished to compare the results of this study to previous UK population studies, which had used the Rutter A scale.^13^ A calibration study was undertaken, which measured both the Rutter A scale and the SDQ scale on an independent sample of adolescents.^10^ These external data were used to impute the Rutter A subscales (for conduct, hyperactive and emotional problems) in the B-CAMHS99 study, but were not included at the analysis stage.

In this scenario, the imputations were generated using extra information that was not utilized by the analyst; i.e. that the target and external data were assumed to be identically distributed. Rubin calls such imputation *superefficient* from the analyst’s perspective.^2^ Superefficient imputations can cause Rubin’s MI variance estimator to be positively biased.^3,5^

## 3 Methods for imputation inference

We describe Rubin’s MI and its modified version, Robins and Wang’s MI and FMB. Robins and Wang have described their complex method for a general missing data setting.^5^ To aid further understanding of this method we restrict ourselves to a more simplified setting, which we describe in Section 3.1. The description of Rubin’s MI and FMB remains the same for a more general missing data setting.

### 3.1 Notation and setup

Suppose *g* random variables Y=(Y1,…,Yg) are intended to be observed on *n* subjects. We use subscripts *i* and *j* to index subjects and variables, respectively, (i=1,…,n; j=1,…,g). Let y=(yij) denote a (*n* × *g*) matrix, whose *i, j* element is *y_ij_*. Row *i* of matrix ***y*** is denoted by yi=(yi1,…,yig). The rows of the matrix are assumed to be independent and identically distributed. In practice, some subjects have missing observations, and we write y=(yobs,ymis) with *obs* and *mis* denoting the observed and missing parts, respectively. In our simplified example, missingness is confined to one variable and without any loss of generality, we assume variable *Y_g_* is incompletely observed for *t* (*t* < *n*) subjects. The MDM is assumed to be ignorable for Bayesian inference,^14, p.120^ and hence is also ignorable for likelihood inference.

Let, ~Y denote *p* − 1 (1≤p-1≤g-1) variables in the set Y1,…,Yg-1 and ~yi the row vector of observations on ~Y for subject *i*. The imputation model is the normal linear regression yig|~yi ~N(~yiμ,σ), where μ is a column vector of dimension *p* − 1. Let θ=(μ,σ)T, a (*p* × 1) vector of unknown parameters.

Let, $y_{\mathrm{ig}}^{k}$ denote the *k*th imputed value of variable *Y_g_* for subject *i*, and $y_{i}^{k}$ is row *i* of the *k*th imputed dataset $yk=(y_{1}^{k},\ldots ,y_{n}^{k})T(k=1,\ldots ,m)$. If *y_ig_* is observed then $y_{\mathrm{ig}}^{k}=y\mathrm{ig}$, and so $y_{i}^{k}=yi$, for all *k*. Let ··Y denote *q* (1≤q≤g-1) variables in the set Y1,…,Yg-1 and $\cdot \cdot y_{i}^{k}$ the set of observations on ··Y for subject *i* of the *k*th imputed dataset. Similarly, $\sim y_{i}^{k}$ is the set of observations on ~Y for subject *i* of the *k*th imputed dataset. Note, in this example, $\cdot \cdot y_{i}^{k}=\cdot \cdot yi$ and $\sim y_{i}^{k}=\sim yi$ for all *k*. In the interest of completeness, we have included the superscript *k*.

The analysis procedure is the normal linear regression model $y_{\mathrm{ig}}^{k}|\cdot \cdot y_{i}^{k}\sim N(\cdot \cdot y_{i}^{k}\beta ,\omega )$, where β is a column vector of dimension *q*. The imputation estimate for the vector of coefficients β=(β1,…,βq)T is denoted by $\beta \wedge I=({\beta \wedge }_{1}^{I},\ldots ,{\beta \wedge }_{q}^{I})T$ and, for j=1,…,q, the variance estimate for ${\beta \wedge }_{j}^{I}$ is ${V\wedge }_{j}^{I}$, with $V\wedge I=({V\wedge }_{1}^{I},\ldots ,{V\wedge }_{q}^{I})T$.

### 3.2 Rubin’s MI inference

Missing values are imputed independently *m* times, to generate *m* imputed datasets. Imputations are drawn independently from the posterior predictive distribution of the missing data given the observed data under a Bayesian model. For k=1,…,m, the analysis model is fitted to each imputed dataset yk separately, generating regression coefficients estimate $\beta \wedge k=({\beta \wedge }_{1}^{k},\ldots ,{\beta \wedge }_{q}^{k})T$ with associated variance estimates ${W\wedge }_{1}^{k},\ldots ,{W\wedge }_{q}^{k}$, where ${W\wedge }_{j}^{k}$ is the variance estimate for coefficient estimate ${\beta \wedge }_{j}^{k}$. The set of *m* coefficient estimates is combined into one MI inference using a simple set of rules. When the estimand of interest is a set of regression coefficients it is simpler to apply these rules separately to each regression coefficient as follows

$${\beta \wedge }_{j}^{I}=m-1\sum_{k=1}^{m}{\beta \wedge }_{j}^{k},\;W¯j=m-1\sum_{k=1}^{m}{W\wedge }_{j}^{k}Bj=(m-1)-1\sum_{k=1}^{m}({\beta \wedge }_{j}^{k}-{\beta \wedge }_{j}^{I})2{V\wedge }_{j}^{I}=W¯j+\frac{m+1}{m}Bj$$

Confidence intervals are based on the Student’s *t*-distribution. When *n* is small a modified degrees of freedom formula is recommended.^15^ The modified degrees of freedom $\nu_{j}^{*}$ is calculated, separately for each regression coefficient, as follows

$$\gamma \wedge j=\frac{(1+m-1)Bj}{W¯j+(1+m-1)Bj},\;\nu j=(m-1)(1+\frac{m}{m+1}\frac{W¯j}{Bj})2{\nu \wedge }_{j}^{\mathrm{obs}}=(1-\gamma \wedge j)(\frac{\nu_{j}^{\mathrm{com}}+1}{\nu_{j}^{\mathrm{com}}+3})\nu_{j}^{\mathrm{com}},\;\nu_{j}^{*}=\{\nu_{j}^{-1}+({\nu \wedge }_{j}^{\mathrm{obs}})-1\}-1$$

where $\nu_{j}^{\mathrm{com}}$ is the degrees of freedom about *β_j_* when there are no missing values.

We shall also consider a modification in which the within imputation variances, $W_{j}^{k}$, are calculated using the robust sandwich variance estimator.^16,17^ This modification only affects the calculation of the variance of the imputation estimate; the imputation estimate remains the same as for Rubin’s MI. We refer to this modified method as ‘robust Rubin’s MI’.

### 3.3 Robins and Wang’s imputation inference

The Robins and Wang variance estimator for imputation does not require multiply imputed datasets, although it may be more efficient when a dataset is multiply imputed.^5^ To our knowledge, there does not exist any commercial or freely available software that calculates this variance estimator. All derivatives involving scalars, vectors and matrices are defined as in,^18^ e.g. for *n* × 1 vectors ***a*** and ***b***, define ∂a/∂bT=[∂ar/∂bc], a square matrix of dimension *n* where *c* and *r*, respectively, refer to column and row numbers.

The *m* sets of imputations are drawn independently from the predictive distribution of the missing data given the observed data under the imputation model evaluated at θ∧, the observed data maximum likelihood estimate of θ. Therefore, each set of imputations is drawn conditionally on the same parameter estimate. In our simple case, θ∧ is the complete case estimate of θ (i.e. estimation is based on the *n–t* subjects with observed *Y_g_*). The *m* imputed datasets are then stacked into a *mn* × *g* dataset (y1,…,ym)T.

The analysis procedure is applied to the stack of imputed datasets (y1,…,ym)T, treating the *mn* rows as independent, and the estimate of β is the Robins and Wang imputation estimate β∧I. The analysis procedure can be non-, semi- or fully parametric such that β∧I is the solution to the estimating equation

$$\sum_{i=1}^{n}m-1\sum_{k=1}^{m}u_{i}^{k}(\theta \wedge ,\beta )=0$$

where $u_{i}^{k}(\theta \wedge ,\beta )=\cdot \cdot y_{i}^{k}(y_{\mathrm{ig}}^{k}-\cdot \cdot y_{i}^{k}\beta )$ for our example.

To calculate the Robins and Wang variance estimator, both the imputer and analyst must generate additional information. The imputer supplies two further datasets based on the score function of the imputation model. The analyst must generate a dataset and a matrix, which are both based on the estimating equation of the analysis procedure evaluated at β=β∧I. The analyst then inputs these pieces of information into a set of matrix formulae to generate V∧I. Below we provide details on how to calculate these pieces of information and the matrix formulae.

The score function of the imputation model is the derivative, with respect to θ, of its log-likelihood function. The contribution from subject *i* to the log-likelihood function is logf(yig|~yi,θ)=0.5(-log2π-logσ-σ-1(yig-~yiμ)2) and its derivative is column vector ∂logf(yig|~yi,θ)/∂θ=[∂logf(yig|~yi,θ)/∂μ,∂logf(yig|~yi,θ)/∂σ]T, where

$$\partial \log f(y\mathrm{ig}|\sim yi,\theta )/\partial \mu =(\frac{y\mathrm{ig}-\sim yi\mu }{\sigma })\sim y_{i}^{T}\partial \log f(y\mathrm{ig}|\sim yi,\theta )/\partial \sigma =\frac{1}{2}(-\frac{1}{\sigma }+\frac{(y\mathrm{ig}-\sim yi\mu )2}{\sigma 2})$$

For subject *i*, when yi is completely observed let $s_{i}^{\mathrm{obs}}=s_{i}^{\mathrm{obs}}(\theta \wedge )=(\partial \log f(y\mathrm{ig}|\sim yi,\theta )/\partial \theta )T|\theta =\theta \wedge$ and when yi is incompletely observed let $s_{i}^{\mathrm{obs}}$ be a zero row vector of dimension *p*. Conversely, for k=1,…,m, when yi is incompletely observed let $s_{i}^{k,\mathrm{mis}}=s_{i}^{k,\mathrm{mis}}(\theta \wedge )=(\partial \log f(y_{\mathrm{ig}}^{k}|\sim y_{i}^{k},\theta )/\partial \theta )T|\theta =\theta \wedge$ and when yi is completely observed let $s_{i}^{k,\mathrm{mis}}$ be a zero row vector of dimension *p*. For each *k* we then have *n* × *p* dataset $S\mathrm{mis},k=(s_{1}^{\mathrm{mis},k},\ldots ,s_{n}^{\mathrm{mis},k})T$ and stacking these *m* datasets we have Smis=(Smis,1,…,Smis,m)T.

For the second dataset based on the score function of the imputation model, first calculate the derivative of column vector ∂logf(yig|~yi,θ)/∂θ with respect to row vector θT, which is the *p* × *p* matrix

$$[\frac{\partial }{\partial \mu T}(\frac{\partial \log f(y\mathrm{ig}|\sim yi,\theta )}{\partial \mu })\frac{\partial }{\partial \sigma }(\frac{\partial \log f(y\mathrm{ig}|\sim yi,\theta )}{\partial \mu })\frac{\partial }{\partial \mu T}(\frac{\partial \log f(y\mathrm{ig}|\sim yi,\theta )}{\partial \sigma })\frac{\partial }{\partial \sigma }(\frac{\partial \log f(y\mathrm{ig}|\sim yi,\theta )}{\partial \sigma })]$$

where

$$\frac{\partial }{\partial \mu T}(\frac{\partial \log f(y\mathrm{ig}|\sim yi,\theta )}{\partial \mu })=-\frac{1}{\sigma }\sim y_{i}^{T}\sim yi\frac{\partial }{\partial \sigma }(\frac{\partial \log f(y\mathrm{ig}|\sim yi,\theta )}{\partial \mu })=-\frac{1}{\sigma 2}\sim y_{i}^{T}(y\mathrm{ig}-\sim yi\mu )=\{\frac{\partial }{\partial \mu T}(\frac{\partial \log f(y\mathrm{ig}|\sim yi,\theta )}{\partial \sigma })\}T\frac{\partial }{\partial \sigma }(\frac{\partial \log f(y\mathrm{ig}|\sim yi,\theta )}{\partial \sigma })=\frac{1}{2\sigma 2}-\frac{(y\mathrm{ig}-\sim yi\mu )2}{\sigma 3}$$

For subject *i* with observed *y_ig_* define

$$d_{i}^{T}=di(\theta \wedge )T=-[n-1\sum_{i=1}^{n}\{\frac{\partial }{\partial \theta T}(\frac{\partial \log f(y\mathrm{ig}|\sim yi,\theta )}{\partial \theta })\}|\theta =\theta \wedge ]-1si\mathrm{obs}(\theta \wedge )T$$

When *y_ig_* is incompletely observed di is a zero row vector of dimension *p*. These *n* row vectors form the second dataset D=(d1,…,dn)T. The imputer’s role is now completed and the stacked imputed datasets (y1,…,ym)T and datasets Smis and ***D*** are passed on to the analyst.

Evaluating $u_{i}^{k}(\theta \wedge ,\beta )$ at the imputation estimate β∧I, the analyst generates *m* (n×q) datasets $Uk=(u_{1}^{k}(\theta \wedge ,\beta \wedge I),\ldots ,u_{n}^{k}(\theta \wedge ,\beta \wedge I))T(k=1,\ldots ,m)$. These *m* datasets are stacked to form dataset U=(U1,…,Um)T. The matrix generated by the analyst is τ=τ(θ∧,β∧I), which is calculated by differentiating column vector $u_{i}^{k}(\theta \wedge ,\beta )T$ with respect to row vector βT, to generate a square matrix of dimension *q*

$$\frac{\partial u_{i}^{k}(\theta \wedge ,\beta )T}{\partial \beta T}=[\frac{\partial u_{i}^{k}(\theta \wedge ,\beta )r}{\partial \beta c}];\;r,c=1,\ldots ,q$$

where *r* and *c*, respectively, denote the row and column of the matrix and $u_{i}^{k}(\theta \wedge ,\beta )r=y_{\mathrm{ir}}^{k}(y_{\mathrm{ig}}^{k}-\cdot \cdot yki\beta )$. We can then calculate

$$\tau =\tau (\theta \wedge ,\beta \wedge I)=-(\mathrm{nm})-1\sum_{i=1}^{n}\sum_{k=1}^{m}(\frac{\partial u_{i}^{k}(\theta \wedge ,\beta )T}{\partial \beta T})|\beta =\beta \wedge I=n-1\sum_{i=1}^{n}\cdot \cdot ykiT\cdot \cdot yki$$

The analyst now inputs datasets D,Smis and ***U*** and matrix τ into the following matrix formulae

$$¯ui=¯ui(\theta \wedge ,\beta \wedge I)=m-1\sum_{k=1}^{m}u_{i}^{k}(\theta \wedge ,\beta \wedge I)\kappa =\kappa (\theta \wedge ,\beta \wedge I)=(\mathrm{nm})-1\sum_{i=1}^{n}\sum_{k=1}^{m}(u_{i}^{k}(\theta \wedge ,\beta \wedge I))Tsi\mathrm{mis},k\Lambda =\Lambda (\theta \wedge )=n-1\sum_{i=1}^{n}d_{i}^{T}di,\;\Omega =\Omega (\theta \wedge ,\beta \wedge I)=n-1\sum_{i=1}^{n}¯u_{i}^{T}¯ui\Delta =\Delta (\theta \wedge ,\beta \wedge I)=\Omega +\kappa \Lambda \kappa T+n-1\sum_{i=1}^{n}\{\kappa d_{i}^{T}¯ui+(\kappa d_{i}^{T}¯ui)T\}\Gamma =n-1\tau -1\Delta (\tau -1)T$$

Finally, for j=1,…,g-1, the *jth* diagonal entry of matrix Γ is the variance estimate corresponding to the coefficient imputation estimator $\beta_{j}^{I}$, i.e. ${V\wedge }_{j}^{I}=\Gamma \mathrm{jj}$.

### 3.4 Full mechanism bootstrapping

FMB can be applied to parametric and non-parametric problems,^8^ and under all three MDM assumptions.^19^ Unless, the MDM is assumed to be missing completely at random (MCAR) then FMB requires modelling of the MDM. In Efron’s worked example of the procedure a deterministic imputation model was used. Shao and Sitter^9^ describe FMB with a random regression imputation method.

FMB is implemented as follows:^8^

1. Impute the incomplete dataset ***y*** once to generate imputed dataset yI. Apply the analysis procedure to dataset yI. The estimate of β is the imputation estimate β∧I.
2. Sample with replacement *n* rows from yI to obtain bootstrapped dataset yI*.
3. Set observations in yI* to missing by applying the same missing data pattern (MDP) as for ***y*** if MCAR is assumed. Otherwise, infer missingness under a model for the MDM.
4. Singly impute the incomplete dataset from step 3, using the same imputation model as in step 1, to generate the bootstrapped imputed dataset yI⊛.
5. Apply the analysis procedure to dataset yI⊛ and store the estimate of β as bootstrap replication β∧I⊛.
6. Repeat steps 2–5 *T* times to obtain *T* bootstrap replicates.

Standard bootstrap formulae^20^ can be applied to the bootstrap replicates β∧I⊛ to calculate the bootstrap variance and confidence intervals for each ${\beta \wedge }_{j}^{I}$. Currently, there does not exist an algorithm or formula for the calculation of the acceleration constant, which is used to generate the bias-corrected and accelerated bootstrap confidence intervals. However, calculating the acceleration constant based on the formula used to construct non-parametric bias-corrected and accelerated confidence intervals can give a reasonable approximation.^8^

## 4 Return to motivating example

We applied the methods of Section 3 to data from the B-CAMHS99 study discussed in Section 2. The data (from the B-CAMHS99 study) consisted of fully observed measurements for sex and the SDQ subscales in 855 (433 boys and 422 girls) adolescents aged 15. The external data consisted of fully observed measurements for sex, the SDQ subscales and Rutter A subscales in 380 (203 boys and 177 girls) adolescents with median age 15 years (interquartile range 13–15 years).

For the purposes of this case study, each Rutter A subscale was imputed separately under an ordinal logistic regression model, with the three SDQ subscales (for conduct, hyperactive and emotional problems) and sex as covariates. The analysis of interest was a linear regression of the Rutter A subscale, with sex and the constant term as covariates, estimated in the B-CAMHS99 study only. We generated 50 imputed datasets for Rubin’s MI, robust Rubin’s MI and Robins and Wang’s MI, and 2500 bootstrap replications for FMB.

Table 1 presents the results of the analyses of the B-CAMHS99 study. As expected, all point estimates were comparable. Robins and Wang’s MI had the smallest standard errors and narrowest confidence intervals for all cases. The maximum percentage difference in the standard errors of Rubin’s MI and Robins and Wang’s MI was just under 23%, and the corresponding Rubin’s confidence interval was 26.5% longer than the Robins and Wang’s confidence interval. In almost all cases, FMB had smaller standard errors than Rubin’s MI, and the maximum percentage difference in the standard errors was just under 14%. The larger standard errors of Rubin’s MI (or robust Rubin’s MI) indicate potential inflation due to superefficient imputations. Robust Rubin’s MI showed little improvement over Rubin’s MI.

**Table 1.** Analysis results of the B-CAMHS99 study using Rubin’s MI (Rubin), robust Rubin’s MI (Rubin R), Robins and Wang’s MI (RW) and full mechanism bootstrapping (FMB).

Scale		*β_cons_*				*β_sex_*			
		Estimate	SE	95% CI	CI width	Estimate	SE	95% CI	CI width
Conduct problems	Rubin	1.13	0.112	(0.910,1.35)	0.441	−0.155	0.158	(−0.467,0.157)	0.624
	Rubin R	1.13	0.110	(0.913,1.35)	0.434	−0.155	0.151	(−0.453,0.143)	0.596
	RW	1.13	0.0968	(0.944,1.32)	0.379	−0.158	0.131	(−0.415,0.0985)	0.514
	FMB								
	Normal	1.09	0.107	(0.883,1.30)	0.420	−0.133	0.147	(−0.421,0.156)	0.577
	Percentile	–	–	(0.911,1.35)	0.437	–	–	(−0.434,0.142)	0.576
	BC	–	–	(0.873,1.28)	0.407	–	–	(−0.393,0.202)	0.595
Emotional problems	Rubin	0.986	0.0887	(0.812,1.16)	0.350	0.291	0.146	(0.00215,0.579)	0.577
	Rubin R	0.986	0.0813	(0.826,1.15)	0.321	0.291	0.141	(0.0121,0.569)	0.557
	RW	0.984	0.0721	(0.842,1.12)	0.283	0.312	0.116	(0.0836,0.540)	0.456
	FMB								
	Normal	1.03	0.0832	(0.862,1.19)	0.326	0.242	0.127	(−0.00565,0.490)	0.496
	Percentile	–	–	(0.823,1.15)	0.326	–	–	(0.0606,0.553)	0.492
	BC	–	–	(0.913,1.24)	0.325	–	–	(−0.0777,0.423)	0.501
Hyperactive problems	Rubin	0.853	0.0863	(0.683,1.02)	0.340	−0.260	0.126	(−0.508,−0.0110)	0.497
	Rubin R	0.853	0.0883	(0.679,1.03)	0.348	−0.260	0.122	(−0.501,−0.0181)	0.483
	RW	0.853	0.0824	(0.686,1.01)	0.323	−0.253	0.107	(−0.462,−0.0433)	0.419
	FMB								
	Normal	0.871	0.0917	(0.691,1.05)	0.359	−0.288	0.119	(−0.520,−0.0551)	0.465
	Percentile	–	–	(0.680,1.04)	0.361	–	–	(−0.490,−0.0229)	0.467
	BC	–	–	(0.723,1.09)	0.369	–	–	(−0.543,−0.0812)	0.462

Imputation estimate with standard error (SE), 95% confidence interval (CI) and CI width for the constant (*β_cons_*) and sex (*β_sex_*) coefficients. Bootstrap confidence intervals: normal approximation (normal), percentile and bias-corrected (BC).

## 5 Simulation study methods

We compared the methods described in Section 3 in four scenarios of incompatibility and misspecification of the imputation and analysis models. The simulation study was based on a hypothetical dataset of one binary variable, sex (0 denoting male and 1 denoting female), and four continuous variables, age, height, weight and natural log of insulin index (hereafter referred to as loginsindex). The data were generated under the following model

(1) sex ~Bernoulli(π), age,height|sex ~N(α0+α1sex,Σ)weight=ι0+ι1sex+ι2age+ι3height+ηsexλ×errorWloginsindex=β0+β1sex+β2age+β3weight+ηsexω×errorL

where *error_W_* and *error_L_* are error distributions and ηsex=1 when sex = 0 and ηsex=η when sex = 1.

Model (equation (1)) was based on a dataset of standard anthropometric measurements of 951 young adults enrolled in the Barry Caerphilly Growth study.^21^ The parameters of the data model were set to the estimates from an analysis of this dataset. The values, to four significant figures, of these parameters were:

π=0.4577,   α0=(25.02,1.774),   α1=(-0.03616,-0.1336),   Σ=(0.55210.0015740.0015740.003705),   ι0=-32.98

ι1=-2.314, ι2=-0.01566, ι3=65.38, λ=12.29, β0=1.854, β1=0.2908, β2=0.08003, β3=0.01119,ω=0.7887 and *η* = 0.5. Different scenarios were created by setting parameters α1,ι1,η and *β*_1_ to their null values; zero vector for *α*_1_, 0 for ι1 and *β*_1_, and 1 for *η*. The values of the remaining parameters were fixed.

The analysis model was the normal linear regression of loginsindex on weight, with adjustment for other variables. The aim of the simulation study was to evaluate the methods with respect to the imputation variance estimator when the imputation estimator was unbiased. To avoid bias in the imputation estimator due to the MDM we set weight to be missing, for a subset of subjects, under an MCAR mechanism. The missing weight measurements were imputed under a normal linear regression model. Both imputation and analysis models included a constant term and assumed that the variance of the error terms was constant for all values of the outcome variable (i.e. homoscedasticity). Unless otherwise stated, error distributions *error_W_* and *error_L_* were normal, weight measurements were missing in men and women and the imputation and analysis models were fitted to the entire sample. The scenarios considered were as follows:

*Scenario 1: Subgroup analysis.* We set the true conditional distributions of age, height, weight and loginsindex to be the same in men and women; i.e. α1=(0,0),ι1=0,β1=0 and *η* = 1. Weight measurements were missing (completely at random) in men only. The covariates of the imputation model were age, height and loginsindex. The covariates of the analysis model were age and weight, and the model was only fitted to the men’s observations. There was incompatibility between the imputation and the analysis model since only the imputation model assumed that the continuous variables were identically distributed in men and women.
*Scenario 2: Heteroscedastic errors.* We set the true conditional distributions of age, height, weight and loginsindex to have different means in men and women; i.e. α1,ι1 and *β*_1_ were set to their non-null values stated earlier. Additionally, among women the variance of weight and loginsindex was set to be 1/4 of the variance in men; i.e. *η* = 1/2. The covariates of the imputation model were sex, age, height and loginsindex, and the covariates of the analysis model were sex, age and weight. The imputation and analysis models were compatible but incorrectly specified because they assumed homoscedastic errors.
*Scenario 3: Omitted interaction.* We set the true conditional distributions of age, height, weight and loginsindex to have different means in men and women. The variances of weight and loginsindex were set to be the same in men and women; i.e. *η* = 1. The covariates of the imputation model were sex, age, height and loginsindex, and the covariates of the analysis model were sex, age, weight and the interaction between weight and sex. The imputation model was correctly specified but because the analysis model included an unnecessary interaction term the imputation and analysis models were incompatible.
*Scenario 4: Moderate and severe non-normality.* This scenario was motivated by a simulation study that investigated the performance of MI methods with non-normal distributions.^22^ Unlike these authors, we were only interested in the effect of the shape of the error distribution, not the size of the error variances. The true conditional distributions of age, height, weight and loginsindex were set to be the same in men and women, i.e. α1=(0,0),ι1=0,β1=0 and *η* = 1. For moderate departures from non-normality, we investigated nine different parameter specifications by setting independently the distributions of *error_W_* and *error_L_* to be the uniform distribution, Student’s *t*-distribution with six degrees of freedom or the log-normal distribution exp{N(0,1/42)}. For severe departures from non-normality, we investigated eight different parameter specifications by setting independently the distributions of *error_W_* and *error_L_* to be the uniform distribution, Student’s *t*-distribution with three degrees of freedom or the log-normal distribution exp{N(0,1)}. All error distributions had mean zero and unit variance. The imputation and analysis models were the same as in the subgroup analysis scenario, although both models were fitted to the entire sample. The imputation and analysis models were compatible, but misspecified because they assumed a normal error distribution.

For all scenarios, we repeated the simulation study for sample sizes *n* = 100 and *n* = 1000 and probabilities 0.6 and 0.4 that weight was observed. For the subgroup analysis scenario only, the probability that weight was observed was one among women and 0.6 or 0.4 among men. For each combination of scenario, parameter specification, sample size and observation probability we generated 2500 independent simulated datasets. Based on 2500 simulations the Monte Carlo standard error for the true coverage probability of 0.95 is √(0.95(1-0.95)/2500)=0.0044,^23^ implying that the estimated coverage probability should lie within the range 0.941 and 0.960 (with 95% probability). For Rubin’s MI and robust Rubin’s MI, we imputed the data using the independent Jeffrey’s prior. For FMB, the data were imputed using the same frequentist imputation method used for Robins and Wang’s MI. Each incomplete dataset was imputed 50 times for Rubin’s MI, robust Rubin’s MI and Robins and Wang’s MI, and 2500 bootstrap samples were generated for FMB.

## 6 Simulation study results

Table 2 presents the results of imputation inference according to the methods of Rubin, robust Rubin and Robins and Wang in the four scenarios of misspecified or incompatible imputation and analysis models, where the probability of observing weight was 0.4. For moderate departure from normality and severe departure from normality, we have reported the results corresponding to the error distributions errorW~exp{N(0,1/42)},errorL~exp{N(0,1/42)} and errorW ~ Student’s *t* with three degrees of freedom, errorL~exp{N(0,1)}. The results for other parameter specifications are summarized in the text below.

**Table 2.** Summary of the simulation results for Rubin’s MI (Rubin), robust Rubin’s MI (Rubin R) and Robins and Wang’s MI (RW) for the weight coefficient: mean bias and empirical variance (Emp. var) of the imputation estimate ${\beta \wedge }_{3}^{I}$, mean of the estimated variance ${V\wedge }_{3}^{I}$, empirical coverage probability (CP) and mean width of the 95% confidence interval for ${\beta \wedge }_{3}^{I}$.

Scenario		*n* = 1000					*n* = 100				
		Mean ${\beta \wedge }_{3}^{I}$	Emp. var ${\beta \wedge }_{3}^{I}\times 1000$	Mean ${V\wedge }_{3}^{I}\times 1000$	CP	Mean width	Mean ${\beta \wedge }_{3}^{I}$	Emp. var ${\beta \wedge }_{3}^{I}\times 1000$	Mean ${V\wedge }_{3}^{I}\times 1000$	CP	Mean width
1. Subgroup analysis	Rubin	0.0112	0.0007	0.0123	0.990	0.014	0.0108	0.0811	0.1350	0.986	0.046
	Rubin R	0.0112	0.0007	0.0122	0.990	0.014	0.0108	0.0811	0.1320	0.984	0.046
	RW	0.0112	0.0007	0.0007	0.948	0.011	0.0108	0.0831	0.0721	0.913	0.033
2. Heterogeneous errors	Rubin	0.0112	0.0126	0.0095	0.909	0.012	0.0111	0.1553	0.1091	0.887	0.042
	Rubin R	0.0112	0.0126	0.0102	0.920	0.013	0.0111	0.1553	0.1146	0.895	0.043
	RW	0.0112	0.0127	0.0130	0.945	0.014	0.0118	0.1729	0.1310	0.863	0.043
3. Omitted interaction	Rubin	0.0112	0.0100	0.0135	0.977	0.015	0.0110	0.1159	0.1528	0.974	0.049
	Rubin R	0.0112	0.0100	0.0135	0.977	0.015	0.0110	0.1159	0.1504	0.974	0.049
	RW	0.0112	0.0100	0.0102	0.952	0.013	0.0117	0.1277	0.1111	0.904	0.040
4. Moderate non-normality	Rubin	0.0112	0.0085	0.0087	0.954	0.012	0.0111	0.0968	0.0955	0.946	0.039
	Rubin R	0.0112	0.0085	0.0087	0.953	0.012	0.0111	0.0968	0.0956	0.947	0.039
	RW	0.0112	0.0086	0.0087	0.947	0.012	0.0118	0.1074	0.0903	0.898	0.036
5. Severe non-normality	Rubin	0.0119	0.0181	0.0092	0.858	0.012	0.0125	0.1377	0.1076	0.880	0.041
	Rubin R	0.0119	0.0181	0.0138	0.918	0.014	0.0125	0.1377	0.1290	0.923	0.044
	RW	0.0119	0.0183	0.0159	0.919	0.014	0.0135	0.1594	0.1198	0.872	0.039

Bootstrap confidence intervals: normal approximation, percentile and bias-corrected (BC). Probability of observing weight was 0.4. Moderate non-normality using errorW~exp{N(0,1/42)} and errorL~exp{N(0,1/42)}. Severe non-normality using errorW~ Student’s t with three degrees of freedom and errorL~exp{N(0,1)}.

First consider the left-hand side of Table 2, corresponding to sample size 1000. The imputation estimates, ${\beta \wedge }_{3}^{I}$, for the subgroup analysis, heteroscedastic errors and omitted interaction scenarios were unbiased; that is, the Monte Carlo 95% confidence interval ${\beta \wedge }_{3}^{I}\pm 1.96\times \sqrt{\mathrm{var}({\beta \wedge }_{3}^{I})/2500}$ contained the true value of *β*_3_. For each of these three scenarios, the mean of the Robins and Wang variance estimate, ${V\wedge }_{3}^{I}$, was close to the sampling variance of ${\beta \wedge }_{3}^{I}$ and the confidence interval coverage probability was close to the nominal level. For the subgroup analysis and omitted interaction scenarios, Rubin’s variance estimates were upwardly biased (i.e. the mean of ${V\wedge }_{3}^{I}$ was larger than the sampling variance of ${\beta \wedge }_{3}^{I}$), leading to conservative confidence intervals that were on average wider than those of Robins and Wang. Conversely, for the heterogeneous errors scenario, Rubin’s variance estimate was downwardly biased and the coverage probability of the confidence intervals was more than 4% below the nominal level. The robust Rubin’s MI method performed similar to Rubin’s MI for the subgroup analysis and omitted interaction scenarios and showed a slight improvement over Rubin’s MI (i.e. higher coverage probability) for the heterogeneous errors scenario.

For the moderate non-normality scenario, the imputation estimate was unbiased and the confidence interval coverage probability was close to the nominal level for all three methods. However, for the severe non-normality scenario, the imputation estimates were upwardly biased for all three methods and for both sample sizes. Robins and Wang MI under-estimated the sampling variance of ${\beta \wedge }_{3}^{I}$ the least and had the highest coverage probability, although this was still 3% below the nominal level. The robust Rubin’s MI method was an improvement on Rubin’s MI such that the confidence interval coverage probability for robust Rubin’s MI was less than 1% below that of Robins and Wang’s MI.

Now consider the right-hand side of Table 2, corresponding to sample size 100. The results for Rubin’s MI and robust Rubin’s MI followed the same patterns as noted for sample size 1000. There was a deterioration in the performance of Robins and Wang’s MI when applied to a dataset of sample size 100. Firstly, the imputation estimates for all but the subgroup analysis scenario were (slightly) upwardly biased. Secondly, the Robins and Wang variance estimates were downwardly biased for all scenarios, resulting in confidence interval coverage probabilities that were at least 3% below the nominal level.

Table 3 reports the corresponding results for FMB. The FMB imputation estimates were unbiased for both sample sizes, and for all scenarios except severe non-normality. However, estimation of *β*_3_ was less efficient than for the other methods. Of the three types of confidence intervals, in almost all cases the percentile confidence interval had the highest coverage probability for the same average confidence interval width.

**Table 3.** Summary of the full mechanism bootstrapping simulation results for the weight coefficient: mean and empirical variance (Emp. var) of the imputation estimate ${\beta \wedge }_{3}^{I}$, mean of the estimated standard error ${V\wedge }_{3}^{I}$ and empirical coverage probability (CP) and mean width of the 95% confidence interval for ${\beta \wedge }_{3}^{I}$.

Scenario		*n* = 1000					*n* = 100				
		Mean ${\beta \wedge }_{3}^{I}$	Emp. var ${\beta \wedge }_{3}^{I}\times 1000$	Mean ${V\wedge }_{3}^{I}\times 1000$	CP	Mean width	Mean ${\beta \wedge }_{3}^{I}$	Emp. var ${\beta \wedge }_{3}^{I}\times 1000$	Mean ${V\wedge }_{3}^{I}\times 1000$	CP	Mean width
Subgroup analysis	Normal	0.0112	0.0108	0.0108	0.952	0.013	0.0111	0.1258	0.1238	0.937	0.043
	Percentile	–	–	–	0.988	0.013	–	–	–	0.981	0.043
	BC	–	–	–	0.858	0.013	–	–	–	0.856	0.043
Heterogeneous errors	Normal	0.0112	0.0146	0.0131	0.933	0.014	0.0112	0.1806	0.1468	0.891	0.046
	Percentile	–	–	–	0.933	0.014	–	–	–	0.910	0.047
	BC	–	–	–	0.934	0.014	–	–	–	0.906	0.047
Omitted interaction	Normal	0.0112	0.0135	0.0137	0.948	0.015	0.0109	0.1559	0.1591	0.940	0.048
	Percentile	–	–	–	0.972	0.015	–	–	–	0.966	0.049
	BC	–	–	–	0.910	0.015	–	–	–	0.906	0.049
Moderate non-normality	Normal	0.0112	0.0104	0.0106	0.950	0.013	0.0113	0.1205	0.1170	0.927	0.042
	Percentile	–	–	–	0.951	0.013	–	–	–	0.938	0.042
	BC	–	–	–	0.950	0.013	–	–	–	0.936	0.042
Severe non-normality	Normal	0.0118	0.0199	0.0176	0.944	0.016	0.0129	0.1648	0.1598	0.928	0.048
	Percentile	–	–	–	0.938	0.016	–	–	–	0.933	0.048
	BC	–	–	–	0.924	0.016	–	–	–	0.912	0.048

Bootstrap confidence intervals: normal approximation, percentile and bias-corrected (BC). Probability of observing weight was 0.4. Moderate non-normality using errorW~exp{N(0,1/42)} and errorL~exp{N(0,1/42)}. Severe non-normality using errorW~ Student’s t with three degrees of freedom and errorL~exp{N(0,1)}.

For the subgroup analysis, heterogeneous errors, omitted interaction and moderate non-normality, when the sample size was 1000 Robins and Wang MI outperformed FMB; i.e. had the narrowest average confidence interval and at least nominal coverage. When the sample size was 100, for the subgroup analysis and omitted interaction scenarios FMB with the percentile confidence interval was marginally better than Rubin’s MI because (for comparable coverage probabilities) the bootstrap confidence intervals were narrower on average than those of Rubin’s MI. The relative inefficiency of FMB to Rubin’s MI was outweighed by the upward bias of Rubin’s variance estimates. For the heterogeneous errors scenario, sample size 100, FMB with the percentile confidence interval had the highest coverage probability, although still outside the expected range (0.941–0.960). For the moderate non-normality scenario, Rubin’s MI was the best method for sample size 100, with close to nominal coverage and the narrowest mean confidence interval width. For the severe non-normality scenario, and for both sample sizes, FMB had the highest coverage probabilities, although still below 0.941.

When the probability of observing weight was 0.6, the pattern of results was the same as in Tables 2 and 3, although the differences between the methods were less marked. Across the nine different error distributions investigated for the moderate non-normality scenario, and for the severe non-normality specifications errorW ~exp{N(0,1)},errorL ~uniform and errorW ~ Student’s *t*-distribution with three degrees of freedom, errorL ~uniform the results were virtually identical to those reported for moderate departures from normality in Tables 2 and 3. Across the remaining six error distributions investigated for severe non-normality, the pattern of the results was very similar to the severe non-normality scenario in Tables 2 and 3, so that the conclusions drawn with respect to the comparisons of the methods remained the same. For the other coefficients of the analysis model, either the pattern of results was the same as in Tables 2 and 3 but with smaller differences between the methods, or all methods had coverage probabilities of 94–95%, with Robins and Wang MI having the narrowest confidence interval on average and FMB having the widest.

For the subgroup analysis and interaction scenarios in several instances, the Robins and Wang variance estimate was smaller than the corresponding variance estimate that resulted from analysing the fully observed data, i.e. data without missing observations (data not shown). This is due to the fact that the imputations are superefficient with respect to the analysis procedure; i.e. the imputations contain extra information that is not contained in the true data.^2^

We conducted a second simulation study to assess the robustness of FMB when data were missing at random (MAR) and the MDM (for simulating missingness in a bootstrapped dataset) was not modelled; that is, missingness was simulated using the observed MDP as above. The design of this simulation study was based on the simulation study described above, with three modifications. There were three changes. First, data were simulated to be missing dependent upon the outcome variable of the analysis model (loginsindex), thus ensuring the complete case analysis produced biased estimates. Second, the imputation and analysis models were compatible and correctly specified. Third, we conducted two FMB methods: FMB with the MDM correctly modelled (which we shall call FMB correct MDM) and FMB with missingness simulated using the observed MDP (which we shall call FMB observed MDP). For both sample sizes, when data were MAR, omitting to model the MDM for FMB resulted in downwardly biased variance estimates, which led to under-coverage of the confidence intervals. Variance estimates were not downwardly biased when the MDM was correctly modelled (FMB correct MDM). For sample size 1000, any differences in the performances of Rubin’s MI, robust Rubin’s MI, Robins and Wang’s MI and FMB correct MDM were very small. For sample size 100, the Robins and Wang method outperformed the other methods with respect to point estimation but its variance estimates were again downwardly biased. For interval estimation only FMB correct MDM with the percentile confidence interval provided coverage probabilities greater than 0.941 for all coefficients. (Results of this simulation study are available upon request from the authors.)

## 7 Discussion

We have conducted the first comparative evaluation of Rubin’s MI, a modified version of Rubin’s MI, Robins and Wang’s MI and FMB. Our simulation study shows that Rubin’s MI variance estimator failed in four common scenarios of misspecification of the imputation and analysis models, and of incompatibility between them. The variance estimates were substantially upwardly or downwardly biased, resulting in confidence intervals that over- and under-covered, respectively. For moderate sample size (*n*=1000) and all scenarios apart from severe non-normality, Robins and Wang’s MI produced the narrowest confidence intervals on average, with close to nominal coverage. When the imputation and analysis models were both misspecified due to severe non-normality all methods had the same biased imputation estimate, but the larger imputation variance estimate of FMB resulted in confidence intervals with coverage probabilities closest to the nominal level.

A key feature of Rubin’s MI is the separation of the imputation procedure from the substantive analysis. This has the advantage that the more technical process of imputation can be done by a specialist, following which multiple analyses can be done on the imputed datasets by non-specialists using standard software. However, this separation may also lead to incompatibility between the imputation and analysis models, when assumptions made during imputation are not carried forward to the analysis stage. For example, estimation for domains (i.e. subgroups or subpopulations) is common for the analysis of survey data and, in particular for large surveys analysed by many users, imputations can be generated ignoring the domain indicator.^24^ In some situations, such as the subgroup analysis and omitted interaction scenarios of our simulation study, incompatibility can in principle be avoided, if the imputer provides sufficient documentation of the imputation model to future users. However, in some instances incompatibility may be unavoidable; e.g. for confidentiality reasons the provider of the imputed data may only disseminate to the users a subset of the observations (or records) used during the imputation stage.^11,25^

Misspecification of imputation and analysis models is a more general problem, which will arise whenever model assumptions are not justified. We investigated two scenarios, in which misspecification was due to heteroscedastic errors and non-normality. Our results suggest that when misspecification arises from heteroscedastic errors, there has to be a sizable difference between the subgroup variances in order for Rubin’s imputation variance estimator to materially under-estimate the sampling variance of the imputation estimator. In this case, heteroscedastic errors in the imputation and analysis models could have been accommodated by conducting separate MI analyses in men and women.

A limitation of the Robins and Wang MI method is that it makes large sample assumptions, which led to downwardly biased variance estimates and confidence interval coverage when applied to small datasets. A major disadvantage of the Robins and Wang method is that calculation of the imputation variance estimate is considerably more complicated than for Rubin’s MI and FMB, with a greater burden placed on both the imputer and the analyst. To our knowledge, there is no generally available software implementing the Robins and Wang method. The analyst must make available derivatives of the estimating equations for use in calculation of variance estimates, and these become harder to calculate as the complexity of the analysis procedure increases. Also, the complexity of the calculations conducted by the imputer increases when there is multiple incomplete variables with a general MDP. For this reason, our simulation scenarios were restricted to data missing in a single variable, as were the scenarios considered in the papers proposing the approach. The Robins and Wang method requires the data to be imputed under a single imputation model. Therefore, currently, it cannot be applied if imputation is conducted using chained equations imputation,^26^ a flexible and commonly used method of imputation that imputes under two or more imputation models. In contrast, calculation of the variance of an imputation estimator by Rubin’s MI method and FMB is straightforward for more complex MDPs and analysis procedures, and can be applied when data are imputed using chained equations imputation.

A limitation of FMB was its inefficiency relative to the other imputation inference methods. Furthermore, FMB requires modelling of the MDM when data are MAR, which is not required by the MI methods of Rubin or Robins and Wang. A further simulation study we conducted showed that FMB required modelling of the MDM when data were MAR. Further work is needed to compare Rubin’s MI with FMB when data are MAR and the missing data model assumed by FMB is incorrectly specified.

In summary, accurate inference requires an unbiased estimator and variance estimator. Rubin’s MI variance estimator may be biased in the presence of incompatibility between the imputation and analysis models and model misspecification. This can lead to over- or under-coverage of confidence intervals. These limitations should be noted in guidelines on the appropriate use of Rubin’s MI,^27^ which should emphasize how incompatibility can be avoided, and the pitfalls that can arise because of model misspecification. The simplicity and flexibility of Rubin’s MI mean that it is likely to remain the method of choice to deal with data that are MAR. However, where these problems of incompatibility and misspecification cannot be avoided, Robins and Wang MI has the potential to provide more robust inferences, should the considerable challenges in provision of software implementing the procedure be overcome.
